# Conversational Task Increases Heart Rate Variability of Individuals Susceptible to Perceived Social Isolation

**DOI:** 10.3390/ijerph18189858

**Published:** 2021-09-18

**Authors:** Ying Xing Feng, Nur Syahirah Roslan, Lila Iznita Izhar, Muhammad Abdul Rahman, Ibrahima Faye, Eric Tatt Wei Ho

**Affiliations:** 1 Centre for Intelligent Signal and Imaging Research (CISIR), Universiti Teknologi PETRONAS, Bandar Seri Iskandar 32610, Perak, Malaysia; nur.syahirah_g03119@utp.edu.my (N.S.R.); ibrahima_faye@utp.edu.my (I.F.); 2 Department of Electrical and Electronic Engineering, Universiti Teknologi PETRONAS, Bandar Seri Iskandar 32610, Perak, Malaysia; lila.izhar@utp.edu.my; 3 Smart Assistive and Rehabilitative Technology (SMART) Research Group, Universiti Teknologi PETRONAS, Bandar Seri Iskandar 32610, Perak, Malaysia; 4 UniKL Royal College of Medicine Perak, Ipoh 30450, Perak, Malaysia; mohammad@unikl.edu.my; 5 Department of Fundamental and Applied Sciences, Universiti Teknologi PETRONAS, Bandar Seri Iskandar 32610, Perak, Malaysia

**Keywords:** heart rate variability (HRV), conversation, extraversion personality, perceived social isolation, mental health

## Abstract

Studies showed that introversion is the strongest personality trait related to perceived social isolation (loneliness), which can predict various complications beyond objective isolation such as living alone. Lonely individuals are more likely to resort to social media for instantaneous comfort, but it is not a perpetual solution. Largely negative implications including poorer interpersonal relationship and depression were reported due to excessive social media usage. Conversational task is an established intervention to improve verbal communication, cognitive and behavioral adaptation among lonely individuals. Despite that behavioral benefits have been reported, it is unclear if they are accompanied by objective benefits underlying physiological changes. Here, we investigate the physiological signals from 28 healthy individuals during a conversational task. Participants were ranked by trait extraversion, where greater introversion is associated with increased susceptibility to perceived social isolation as compared to participants with greater extraversion as controls. We found that introverts had a greater tendency to be neurotic, and these participants also exhibited significant differences in task-related electrodermal activity (EDA), heart rate (HR) and HR variability (HRV) as compared to controls. Notably, resting state HRV among individuals susceptible to perceived loneliness was below the healthy thresholds established in literature. Conversational task with a stranger significantly increased HRV among individuals susceptible to isolation up to levels as seen in controls. Since HRV is also elevated by physical exercise and administration of oxytocin hormone (one form of therapy for behavioral isolation), conversational therapy among introverts could potentially confer physiological benefits to ameliorate social isolation and loneliness. Our findings also suggest that although the recent pandemic has changed how people are interacting typically, we should maintain a healthy dose of social interaction innovatively.

## 1. Introduction

Social relationships are crucial for well-being [1] as human societies are built on a dynamic and complex system with frequent interactions between individuals [2]. Living alone, having few social network ties and paucity of social contact are common markers of social isolation [3,4]. Social isolation is a risk factor for depressive symptoms in early adolescence [5]. Both objective isolation and loneliness (perceived isolation) were associated with increased mortality [3]. The degree of loneliness is also associated with severity of depressive symptoms, regardless of age, gender, ethnicity, education, income, marital status, social support and stress levels [6]. Unintended social isolation and long-term loneliness lead to poorer cognitive function [6] and increased risk of early mortality [1,3,4], consistent across time, genders and geographically [4].

An earlier study found that social and emotional loneliness were inversely related to extraversion [7], whereby literatures had contradictive findings on whether men [7] or women [8] have greater susceptibility to loneliness. A latest meta-analysis (N = 93,668) [9] confirmed that extraversion trait has the strongest association with loneliness (negatively correlated), followed by neuroticism (positively correlated) and other minority traits in the big five personality scales. Children with autism spectrum disorder (ASD) also exhibited reduced trait extraversion, accompanied by higher trait neuroticism [10], which suggests a strong association between reduced extraversion with ASD and social isolation. Among the healthy population, introversion refers to the non-clinical part of autism spectrum varying in degrees of severity in expression, despite their apparent synonymous nature of traits [11]. Introvert students reported that social media are more helpful to boost learning performance and self-confidence because face-to-face contact hampered their true nature [12]. A recent study confirmed that extraversion trait (rather than neuroticism) is the dominant predictor of forecasted affect in relation to social situations [13]—that introverts are more likely to reduce their approach behavior in social situation due to undesirable expectation. These studies indicate that individuals with different extraversion traits possess a different propensity towards perceived social isolation.

We believe that humans are innately social, including introverts. Difference in extraversion traits should indicate our social preferences (offline vs. online) rather than the degree of social desires. Often, lonely individuals turn to social media for social support, to compensate their needs and social disadvantages (e.g., awkwardness, interpersonal skills) in face-to-face settings [14]. However, social media may not be a straightforward solution because it could do more harm than good. A study on depression and social isolation reported that frequent internet usage leads to poorer interpersonal relationships among adolescents [15], and the use of Facebook specifically was negatively associated with self-reported mental health [16]. Perceived social isolation also contribute to declined cognition and heightened sensitivity to social threats, which hinder interpersonal interaction, leading back to heightened loneliness and morbidity [17] eventually. These could be the reason why introversion trait has the strongest association with loneliness among other personality traits [9]. Taken together, introverts—who are more susceptible to perceived social isolation—are likely to seek comfort through social media, which, however, hinder their interpersonal skills and mental health. We need an immediate intervention to break the loop, because the negative impact of reliance on social media towards mental health is far greater than the positive impact attained through offline interactions [16].

Verbal interaction is an important social behavior in many vertebrate species, including our own [18]. Female children who interacted with their mother in person or over the phone after social stressors showed reduced levels of salivary cortisol, while subjects who texted their mothers showed heightened levels of salivary cortisol similar to control subjects who did not interact with their parents at all [19]. Additionally, children who received comforting speech alone showed a strikingly similar hormonal profile of elevated oxytocin and swift return of salivary cortisol to baseline, comparable to children who were comforted through physical, vocal and non-verbal means [18]. Both studies [18,19] suggest that dialogue with their loved one may be as important as touch due to neuroendocrine regulation of social bonding in our species. Remarkably, a study of 42 blind veterans who received group therapy by phone calls to treat affective disorder related to isolation [20] reported a significantly lowered level of loneliness and increased social activities. Socially isolated college students who were assigned to social interaction practice also showed significant reduction in social anxiety and increased social activity, where such benefits were consistent during a follow-up assessment 15 months later [21]. These studies indicate the psycho-physiological benefits of verbal interaction do not come explicitly from close contacts, but also from acquaintances such as social workers or college mates.

Contrary to the traditional belief that introverts require less social stimuli and enjoy having alone time, a recent study [22] found that a higher degree of introversion trait predicts heightened depressive symptoms along with a higher anxiety and loneliness experience following the implementation of social distancing and lockdown measures to curb the spread of COVID-19 pandemic. Alleviating social isolation and loneliness became an important area for policy and practice, but the effectiveness of many interventions has been questioned due to insufficient evidence [23]. A meta-analysis on studies involving adolescents and adults (N = 787) found that negative social interactions decreased HRV (i.e., similar effects when undergoing the Trier Social Stress Task), but typical or positive dyadic social interactions do not increase HRV from the baseline level [24]. A later review [25] also indicated the absence of a clear, effective intervention for social isolation and loneliness. In this study, we investigate the physiological evidence underlying the rewarding nature of social interaction [2] through a verbal communication paradigm. We used conversational task [26] as a proxy for social interaction. Participants were asked to share their ideas casually to a stranger (same human across study) who was trained to listen to the participants actively and provide an agreeable neutral expression throughout the session. We conducted the study in one-on-one laboratory settings to avoid unnecessary anxiety or stress responses which resemble public speaking.

We measured the respiration rate (RESP), skin conductance (EDA) and blood volume pulse (BVP that reflects HR and HRV), and subsequently compared each measure between talking (TASK) and resting state (REST) due to superior temporal resolution of these physiological signals. Besides, post-experiment lab analysis was not required, unlike salivary cortisol and oxytocin hormone samples. Physiological changes such as increased respiratory activity, cardiac activity, or electrodermal activity (EDA) indicate heightened emotional [27] and psychological arousal [28]. An optimum level of heart rate variability (HRV) is associated with good health, self-regulatory capacity, adaptability and resilience [29]; whereby regular physical activity can also increase HRV in aging subjects [30]. HRV can also predict both morbidity and mortality [31]. We hypothesized that verbal interaction induces changes in HR and are particularly interested to discover if verbal conversation could yield similar interventive effects to increase HRV, apart from the established benefits in alleviating the feeling of perceived social isolation. We seek to understand (1) if the effects of conversational task could be observed and measured through physiological markers and (2) if extraversion personality traits appear as an important modulating factor on changes in these physiological signals.

## 2. Materials and Methods

### 2.1. Participants

We received a total of 91 complete applications among healthy male university students aged between 18–21 years old through an online recruitment. All participants were asked to complete the Big Five Inventory (BFI) and Eysenck’s Personality Inventory (EPI) tests online; 41 participants who completed the online tests and showed consistent extraversion trait in BFI and EPI were invited to participate in the experimental session. Given the available timeframe, 28 participants (mean age ± SD: 19.50 ± 0.76 years) completed the study successfully (turnout rate 68%).

According to self-reports, none of the participants were suffering from, or had a family history related to a cognitive disorder, nor have taken any illegal drug or under prescribed medication (within a week). None of them reported sleep deprivation nor experienced adverse psychological experience prior participating in this study.

### 2.2. Personality Assessments

To investigate if personality traits act as a mediating factor for physiological evidence during conversation task, we administered 2 personality tests to assess the participant’s tendency of extraversion, namely the BFI [32] and EPI [33]. Extraversion score for EPI ranged from 1 (highly introvert) to 24 (highly extrovert), while BFI ranged between 1 (highly introvert) to 5 (highly extrovert). The median score for extraversion in EPI and BFI were 12.5 and 3, respectively. To eliminate confounding factor due to uncertain extraversion trait, participants who scored between 11 to 13 in EPI, 3 in BFI or showed inconsistent traits in both tests were excluded. The participants who voluntarily showed up in both groups were coincidently balanced: introverts (N = 14) and extroverts (N = 14).

### 2.3. Conversational Task

Participants were asked to be seated in a comfortable chair in a sound-attenuated and temperature-controlled room with adequate lighting (warm white). Prior to giving the written consent, they were briefed about the purpose and flow of the experiment. Physiological sensors were then affixed for baseline recordings in eyes-closed and eyes-open conditions, 5 min for each session. Next, participants were asked to make eye contact with the inquirer (a stranger, see settings in Figure S1). Participants were then asked to share their thoughts verbally based on the topics raised by an inquirer while the physiological signals were measured simultaneously. We referred to a seminal work [26] which introduced a new flexible speech task based on simple question and answering sessions. To begin with, we asked the participants to briefly introduce themselves (2 min) followed by four questions with regards to general topics in life (2 min each, questions in Text S1) without any prerequisite nor preparation needed. For timekeeping, once the time is up for each question, they were asked to stop immediately and move on. During the debrief session, physiological sensors were removed, and the participants were asked to share their experience in helping the researcher to identify if any distress occurred throughout the study. None of the participants described the session as tense or unpleasant. Lastly, they were refrained from sharing the discussed topics with their peers so that consistency remained across participants.

### 2.4. Physiological Assessments

FlexComp Infiniti Encoder (Thought Technology Inc., Montreal, QC, Canada) was used to assess the participants’ autonomic nervous system response non-invasively through heart activity, electrodermal activity (EDA) and RESP pattern. The EDA was measured by a dual-electrodes (part number: SA2659) skin conductance sensor (SC sensor, model SA9309M) adhered on the inner tips of index and ring fingers; while the heart activity was measured by a blood volume pressure (BVP sensor, model SA9308M) placed on the middle finger. RESP was measured using the respiration sensor (model SA9311M) that attached to high durability woven elastic band, positioned right below the outer chest line of the participant (see complete setup in Figure S2). SC and RESP were sampled at 256 Hz; while for BVP was sampled at 2048 Hz for a continuous record of the participant’s physiological responses. The complete technical specifications of each sensor can be obtained through (http://www.thoughttechnology.com/sciencedivision/pages/products/ (accessed on 17 February 2020)). Prior data acquisition, we ensured that the sensors were placed correctly by checking the signals’ quality in each participant.

### 2.5. Data Analysis

The statistical data for all physiological features were extracted using the BioGraph Infiniti Software (Thought Technology Inc., Montreal, DC, Canada) before being exported to MATLAB (The MathWorks, Inc., Natick, MA, USA) for further analysis. The EDA was represented by mean skin conductance in microsiemens (µS); while mean heart rate (beats/min) was computed based on the volume of blood that passes through the tissues in a localized area with each beat (pulse) of the heart through the BVP sensor. For mean RESP rate (breath/min), the readings were recorded by counting the number of times the chest rises or falls per minute. Indirect features such as the BVP amplitude (mV), HR peak-to-trough (HR max–HR min, HRmm) and standard deviation for HR inter-beat interval (IBI) known as SDRR were also extracted via BioGraph Infiniti. BVP amplitude was estimated based on the averaged R spike amplitude multiplied with 0.15 mV resolution over a 16-bit ADC. HRmm is a feature of HRV that is most likely used in clinical settings [34]; whereas SDRR obtained through BVP is the proxy for HRV-SDNN via chest ECG [35].

Prior to statistical analysis, we screened the individual bio-signals’ plot to eliminate participants with incomplete or contaminated data. Data which showed drastic increase or decrease (>±2 SD) from the mean value (averaged session) were rejected as motion artifact. Such changes could be due to swift detachment of the sensor during vigorous bodily movement. The binary task engagement states were defined as REST (eyes-open resting state, 5 min) and TASK (actively talking, 10 min in total), respectively. We performed normality tests on all the physiological parameters using SPSS (IBM Corp., Armonk, NY, USA) and adapted non-parametric tests to examine if the population data were significantly different (a paired comparison using Wilcoxon signed-rank test) between binary engagement states (mean values in TASK vs. REST). To further examine if the difference between extraversion groups is significant, we used Mann–Whitney U-test (unpaired) to compare each physiological marker during resting state and task state disjointedly. We also repeated the Wilcoxon ranked test (paired) to examine if the earlier statistical evidence for binary engagement states contributed differently by each group (introverts and extroverts). Lastly, the Cohen’s d equivalent effect size (η^2^) for non-parametric test was estimated based on the Wilcoxon z-value and sample size [36].

## 3. Results

### 3.1. Extraversion Personality Indexes

The mean and standard deviation (SD) extraversion scores for extroverts and introverts were tabulated in Table 1. The personality scores related to extraversion between both groups were significantly different in both EPI (t_(26)_ = 12.311, *p* < 0.001) and BFI (t_(26)_ = 6.889, *p* < 0.001).

We also found the majority introverts (10 out of 14) had neurotic trait based on the EPI score (neuroticism index > 12), as compared to extroverts (6 out of 14). However, none of the extroverts showed similar neuroticism tendency in BFI (index > 3) unlike a few introverts. Interestingly, the remaining 4 introverted participants who are close to the borderline of neuroticism trait (index 9~12) showed the highest lie score in EPI (see data in Table S1).

**Figure 1:**
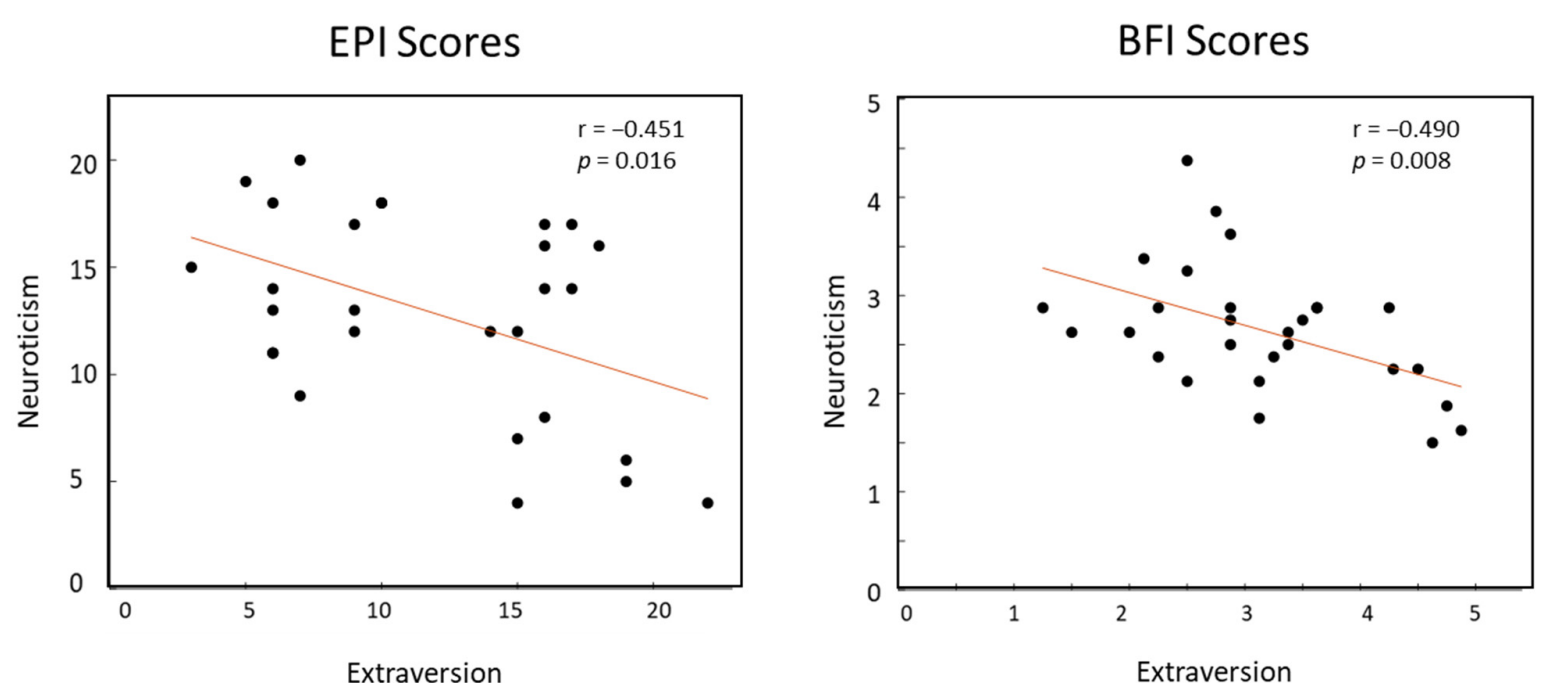
Correlation between neuroticism and extraversion index through EPI (**left**) and BFI (**right**) scores.

Despite that categorical neuroticism is not consistent between extraversion groups and across tests, we found moderate negative correlation (*p* < 0.05) between neuroticism and extraversion traits, and it is consistent across EPI and BFI scores as shown in Figure 1.

### 3.2. Physiological Responses for Binary Task Engagement States

**Figure 2:**
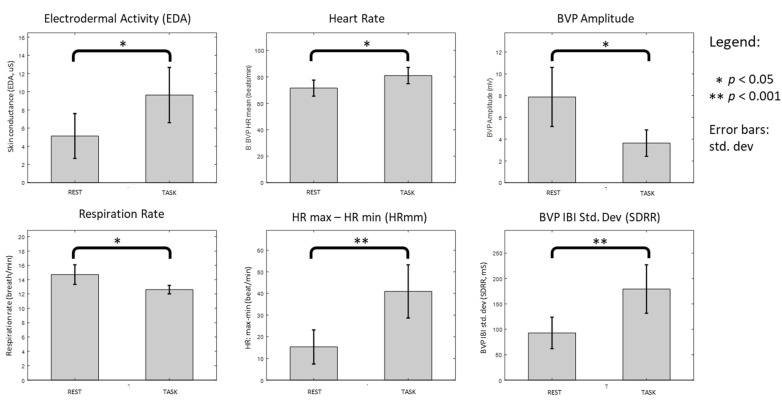
Physiological markers during speech task (TASK) were significantly different as compared to baseline (REST).

The statistical output from the Shapiro–Wilk test, histograms, normal Q-Q plots, box plots as well as skewness and kurtosis values indicate that part of the physiological markers’ data did not meet the assumptions for parametric test. We opt for multiple comparisons using the non-parametric test as an alternative. The mean and standard deviation (SD) for each parameter were represented using bar graphs with extended error bars, as shown in Figure 2.

Wilcoxon signed-rank tests showed significantly higher EDA (*Z* = 2.993, *p* = 0.00276), mean HR (*Z* = 2.803, *p* = 0.00507), HRmm (*Z* = 4.134, *p* = 0.000036) and SDRR (*Z* = 3.737, *p* = 0.000186), and significantly lower BVP amplitude (*Z* = −3.183, *p* = 0.00146) and mean Resp (*Z* = −2.736, *p* = 0.00622) during TASK as compared to REST.

**Figure 3:**
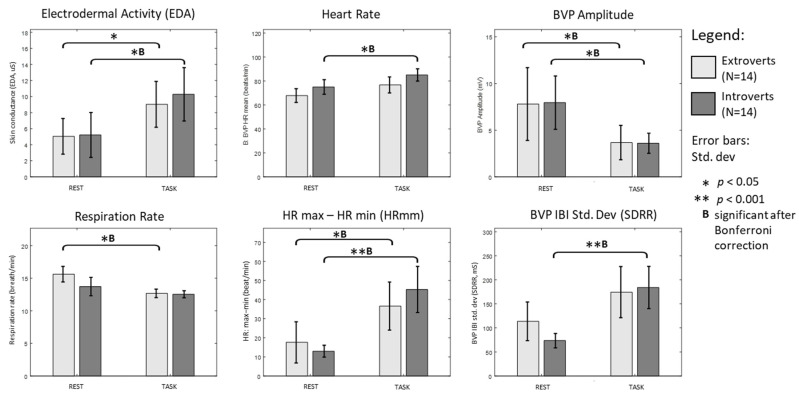
Changes of physiological signals between REST-TASK states are modulated by trait extraversion.

We compared the physiological responses between introverts and extroverts in Figure 3. In both REST and TASK engagement states, Mann–Whitney U-tests did not reveal significant differences between introverts and extroverts. However, the analysis suggests that physiological differences between TASK and REST were predominantly prominent among introverted participants.

Introverts showed significantly higher EDA, HR, HRmm, HRV-SDRR and lower BVP amplitude during TASK; while extroverts showed significantly higher HRmm, lower BVP amplitude and lower RESP during TASK (Wilcoxon tests, *p* < 0.05). Most of these statistical inferences between binary engagement states hold even after Bonferroni corrections were applied for multiple comparisons (α = 0.05/2).

EDA of extroverts indicates a higher skin conductance during TASK, but the difference was no longer significant after Bonferroni correction is applied. Physiological parameters such as HR and SDRR among extroverts did not reveal difference between binary engagement states statistically; whereas the RESP rate between TASK and REST among introverts failed to reject the null hypothesis. Data for all statistical tests in Figure 3, including the estimated effect size is showed in Table 2. 

## 4. Discussion

### 4.1. Personality Traits and Perceived Social Isolation

Introverts in this study are likely to have neuroticism trait (71.4% in EPI), making them more susceptible to experience perceived social isolation (loneliness). A study found that neurotic introverts have a greater risk of psychological distress syndromes and undesirable social outcomes [13] which demotivate their intention to socialize. Interestingly, the remaining introverts who showed marginal neuroticism score had the highest EPI lie score, suggest an intention to showcase themselves differently [37]. We also found significant negative correlation between extraversion and neuroticism in both EPI and BFI tests, supporting theories which associate introversion to loneliness. fMRI study revealed that loneliness is positively correlated with neuroticism and negatively related to extraversion trait, and these relationships were also reflected in changes of gray matter volume in the left dorsolateral prefrontal cortex [38]. Although both introversion and neuroticism are significantly correlated with social loneliness [7], later studies had confirmed that introversion trait had the strongest association with loneliness [9] and predominantly predicts the forecasted affect in social situations as compared to neuroticism [13].

### 4.2. Conversational Task Broadly Modulates Physiological Signals

Respiration rates during TASK and REST were within the range for healthy adults (12 to 18 breaths per minute) [39]. Although emotional arousal or physical activity may induce faster and deeper respiration [40], we found decreased respiration rates instead in both groups during TASK. We postulate that finite lung capacity among participants may require an extended exhalation to support verbal articulation [41] during the conversational task. Significantly lower respiration among participants with higher trait extraversion is likely associated to their verbose nature.

EDA measurements obtained from both extraversion groups during both states are within the established range (2–20 μS) of hypothetical skin conductance recordings [42]. EDA for both groups increased in response to cognitive and affective stimuli [28,43] which reflects elevated affective-arousal state [44] during TASK, particularly among the introverts who may not favor an actual social situation [13].

We obtained comparable HR (mean: 77.5 ± 16.3 beats/s) with a physiological study that employed a similar measuring device on healthy subjects [28]. In contrast to an early work [45], which studied impromptu speech task, reporting only elevated HR, our data showed a significant increase in both HR and HRV (SDRR and HRmm) during conversational task. Our findings on HRV also opposed to study that suggest significantly increased HR and decreased HRV is associated with acute psychological stress [46].

We found a significant lower BVP amplitude during TASK, which is across extraversion traits. Our findings correspond to a study that found a significantly lower BVP amplitude among securities traders during live trading, across all stock market volatility events and regardless of experience [47]. Significantly higher BVP amplitude, on the other hand, is reported among cancer patient caregivers who were given different relaxation interventions on-duty [48]. These findings suggest that BVP amplitude could be a reliable marker to distinguish task engagement states in various settings.

Conversational task with a stranger significantly elevated the overall population mean HR, HRmm, SDRR, EDA and decreased BVP amplitude and RESP. Modulation of many physiological signals suggests that conversational task is engaging. Elevated EDA and lower BVP amplitude is associated with the activation of systemic nervous system [49] while higher HR and HRV is linked to increased cognitive effort [50] and positive effects of regular exercise [30], rather than indicating psychological stress [46]. Based on these findings, it is likely that participants do not perceive our conversational task as stressful, particularly the extroverts.

### 4.3. Physiological Signals Are Modulated Differently by Trait Extraversion

Physiological measures such as HR, HRmm, SDRR and EDA were modulated differently by extraversion trait. HRmm and EDA in individuals with propensity towards perceived social isolation showed greater elevation during conversational task as compared to controls. Elevated EDA, HR and reduced BVP amplitude is linked to arousal of the sympathetic nervous system (SNS) [49,51] and was also observed in autistic children while performing a mental-related task [52]. In contrast, EDA modulations between REST and TASK in the extroverted group were not significant after Bonferroni correction.

HR only changed significantly from REST to TASK among introverts, which suggests that individuals with propensity towards isolation perceived conversational task as a more challenging and engaging task [53], compared to extroverted controls. Our findings are also consistent with earlier studies which established differences in response to stress among subjects performing a speech task [54] and elevated HR in subjects with social phobia during impromptu speech [45]. These findings suggest that although introverts may perceive the conversational task rather challenging, the physiological patterns were different than established findings [46] that indicate stress per se.

Prior to conversation, introverts showed depressed HRV-SDRR. The mean HRV-SDRR for extroverts and introverts during REST were 113.44 ms (healthy) and 73.40 ms (compromised health), respectively. Reduced HRV is an indicator of early mortality [31] and is associated with increased cardiac risk [55]. Subjects with HRV over 100 ms had 5.3 times lower risk of mortality compared to subjects who scored under 50 ms [31]. Among introverts, HRV-SDRR rapidly escalated to levels comparable to extroverts during conversational task (mean: 183.83 ms ± 43.89, *p* < 0.001). Similar physiological modulations were observed upon administration of oxytocin [56,57] and with regular physical exercise [30,58]. Our findings suggest a possible utility of conversational task to enhance HRV and potentially, its attendant health benefits.

We found lower HR in extroverted controls during TASK and REST which suggests that they find the conversational task and meeting a stranger less stimulating. HR is an index for vagal tone and reflects autonomic brainstem regulation to restore homeostasis in response to stress [59]. Extroverted controls also showed a higher resting-state HRV-SDRR. This pattern is associated with greater tendency for social engagement as was found in a study of the effects of oxytocin [57], a nonapeptide hormone and neurotransmitter released during social interaction and contact [60]. An animal model study on dogs also showed reduced HR and increased HRV after administration of oxytocin [61].

### 4.4. Conversational Task Is a Potential Therapy for Improving HRV

Although being with friends [62] and having verbal communication with someone trusted can reduce stress [19], we found that conversation with a stranger can also yield similar physiological effects. Interaction with a person rather than merely verbal articulation appears to be crucial because earlier study on reading aloud, solo speech and verbalizing mental arithmetic produced only minor differences in HRV [63]. Unlike introverts, we found no significant increase in HRV-SDRR among extroverted controls during TASK, in line with a meta-analysis (N = 787) which found that typical or positive dyadic social interactions do not increase HRV from the baseline level [24]. Such findings are noteworthy as significant increased HRV-SDRR among introverts per se suggests that the beneficial outcome of conversation is more pronounced among those who are more susceptible to perceived social isolation. Therefore, we postulate that conversational task could potentially be an effective intervention to increase HRV—that forestall the negative effects of perceived social isolation.

The evolution of heart rate monitors in the past two decades has made it possible to measure the effect of therapies on HRV effortlessly [58]. There is a growing body of research on the health benefits of training protocols to improve HRV [64], but little has emerged in the conversational paradigm. Our results point toward the possibility of applying conversational therapy as a non-chemical intervention to improve HRV. Particularly, we are interested in its potential as an intervention for children with autism spectrum disorder. Children with autism spectrum disorder manifest traits similar to extreme introversion personality [65] including poor speech development. HRV could be used as a non-invasive biomarker through portable monitoring devices to evaluate the efficacy of social and conversational therapies [66]. Our findings also suggest that conversation with someone is important to mitigate the risk of declining mental health during the current pandemic [67], particularly among introverts [22].

## 5. Conclusions

In this study, we established that physiological markers effectively distinguish active conversation from resting state. Conversational task with a stranger also produced different physiological effects among participants with distinct extraversion tendency. Introverted individuals showed highly significant elevation of HRV—a biomarker that signify therapeutic effects in physical exercise and direct administration of oxytocin (hormone released during social bonding). Since the COVID-19 pandemic has changed and elevated the importance of conversation, our findings suggest that talking to an acquaintance while keeping a safe distance in the neighborhood is beneficial, particularly among introverts or those living alone during such difficult times.

### Limitation and Recommendation

We suggest interpreting earlier studies related to social isolation differently due to the current advancement in technology and outpouring of social media platforms. The well-being of lonely individuals can be enhanced through social media by increasing self-disclosure (e.g., status updates, sharing of photos) and expecting social support from their friends (e.g., replies, comments) in return [68]. Connecting to social media also benefited minority individuals with certain health conditions, especially if they are geographically isolated [69]. Whilst common dogma viewed that technology can ameliorate social isolation and loneliness, a contradicting study found that young adults who spent the most time or frequency on social media are up to 3 times more likely to perceive themselves as socially isolated [69]. Meta-analysis (N = 8798) reaffirmed that internet usage is positively associated with loneliness [14], apart from being significantly associated with increased depression [70] reported in large-sample study (N = 1787). We believe that perceived social isolation (i.e., loneliness) are determinants of how an individual interacts with the digital world (see [71]), rather than how frequently the individual spends time on social media per se. When technologies are used to escape the social discomfort in actual setting, feelings of loneliness increased [72]. Besides, perceived social isolation predicts various complications above and beyond what is predicted by objective isolation [73]. Therefore, future studies related to social isolation should take an individual’s digital footprint into consideration.

We obtained the HRV-SDRR through BVP sensors placed on the fingers due to limitations in facility and cultural sensitivity. SDRR may include abnormal beats and thus lacks precision as compared to SDNN (normal-to-normal beat) derived from chest ECG. Although the HR derived from both ECG and BVP are highly correlated [35], we encourage future study to consider chest ECG in order to reaffirm our findings, especially if the recording time became shorter [29].

One major limitation of our exploratory study is that we recruited male-only participants, so our findings might be limited to young adult males. An established study (N = 276) on healthy adults (24 h measure) found no significant gender difference in various HRV parameters that signify vagal modulation [74], except for the standard deviation of average NN intervals which were higher among men. Remarkably, such gender difference is diminishing with age linked to reduced hormone estrogen in women; whereas men with higher body mass index showed lower HRV. A clinical study [75] also revealed that all short-term HRV time domain indices had no significant gender difference among healthy controls, except for asthmatic patients. Taken together, gender may have minor or no effect on different HRV indices among healthy adults where such effects were anticipated to diminish with age. Future study should then be aware of potential confounding factors such as gender, age and health status on HRV findings.

Our study also lacks objective assessments to (i) quantify the degree of social desire (i.e., loneliness), (ii) understand how participants are interacting with the digital world and (iii) understand their current relationship status and demography. Although studies had established that higher introversion is associated with higher loneliness and depression, collecting such data may help to better address why introverts are prone to perceived social isolation, along with if our desire for social bonding and affective well-being are predictive through physiological markers. Lastly, it is also important to consider the participants’ ability to focus for a longer duration, interest in participating and ethics reasoning while striving for comprehensiveness in future study.

## Figures and Tables

**Table 1:** Extraversion personality scores between extroverts and introverts.

Extraversion Personality Scores	Extroverts	Introverts
(Mean ± SD)		
Big Five Inventory (BFI)	3.88 ± 0.64	2.37 ± 0.52
(score range: 1~5)		
Eysenck’s Personality Inventory (EPI) (score range: 1~24)	16.79 ± 2.12	7.07 ± 2.06

**Table 2:** Wilcoxon signed-rank tests between REST and TASK for extroverts and introverts.

Physiological Data	Extroverts	Introverts
EDA	Z = −2.045, *p* = 0.0409 *, η^2^ = 0.299	Z = −2.256, *p* = 0.0241 *, η^2^ = 0.364
Heart rate	Z = −1.846, *p* = 0.0649, η^2^ = 0.243	Z = −2.274, *p* = 0.0229 *, η^2^ = 0.369
BVP amplitude	Z = 2.256, *p* = 0.0240 *, η^2^ = 0.364	Z = 2.274, *p* = 0.0229 *, η^2^ = 0.369
RESP	Z = 2.872, *p* = 0.00408 *, η^2^ = 0.589 ^	Z = 0.895, *p* = 0.371, η^2^ = 0.0570
HRMM	Z = −2.281, *p* = 0.0225 *, η^2^ = 0.372	Z = −3.724, *p* = 0.000196 **, η^2^ = 0.991 ^^
SDRR	Z = −1.436, *p* = 0.151, η^2^ = 0.147	Z = −3.929, *p* = 0.0000855 **, η^2^ = 1.103 ^^

Notes: Significant: * *p* < 0.05, ** *p* < 0.001; Equivalent effect size: ^ η^2^ > 0.5 (medium), ^^ η^2^ > 0.8 (large).

## Data Availability

The data presented in this study are available on reasonable request from the corresponding author. The data are not publicly available due to privacy or ethical considerations.

## Supplementary Materials

The following are available online at https://www.mdpi.com/article/10.3390/ijerph18189858/s1, Figure S1: Settings for conversation in a closed room, Text S1: Topics raised by the inquirer, Figure S2: Setup for physiological measures on participants, Table S1: Participants’ personality scores.
