# Supplementary material for: Conversational Task Increases Heart Rate Variability of Individuals Susceptible to Perceived Social Isolation
Source: Int J Environ Res Public Health. 2021 Sep 18;18(18):9858. doi: 10.3390/ijerph18189858 (PMC8466201; doi:10.3390/ijerph18189858)
Supplement: Supplementary file 1 [file ijerph-18-09858-s001.zip › ijerph-1349791-supplementary.pdf]

Supporting Information

Figure S1. Settings for conversation in a closed room

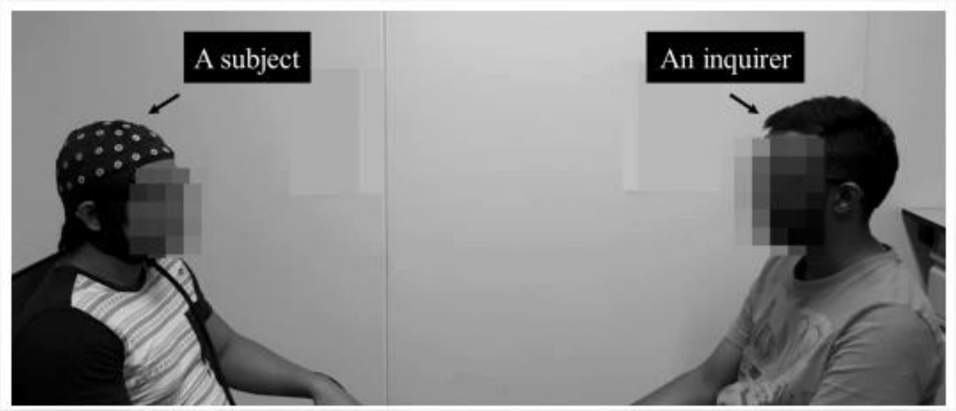

Text S1. Topics raised by the inquirer

1) What song or movie describes you, why?
2) How was your transition from high school to university? What problem (one) did you faced, and how did you handle it?
3) How would you describe the color "yellow" and “red” to a blind (since born)? Discuss how you came across the idea?
4) If you were given $1 million, what would you do with it?

Figure S2. Setup for physiological measures on participants

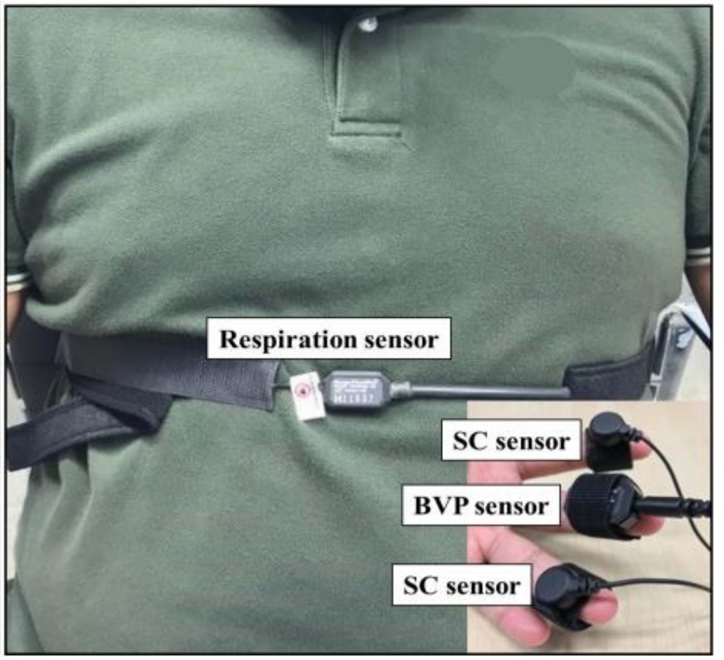

Table S1. Participants' personality scores.

				EPI scores			BFI scores (1-5)				
Old ref	Subject ID	AGE	E/I	L (1-9)	E (1-24)	N (1-24)	E	A	C	N	O
V002	S1	20	E	2	14	12	3.38	4.56	3.33	2.63	3.60
V003	S4	20	E	0	16	14^	4.25	4.33	2.56	2.88	3.10
V020	S7	20	E	4	18	16^	3.50	5.00	3.11	2.75	3.30
V030	S8	19	E	3	15	7	4.29	3.11	2.89	2.25	3.80
V032	S9	21	E	4	19	6	4.75	4.56	3.00	1.88	4.60
V044	S10	20	E	3	19	5	4.63	3.67	3.56	1.50	4.10
V049	S11	19	E	2	22	4	4.88	3.78	2.67	1.63	4.30
V045	S12	20	E	2	17	14^	3.25	3.56	2.78	2.38	3.90
V062	S14	19	E	3	16	8	4.50	4.67	3.44	2.25	3.50
V080	S21	19	E	5*	15	12	3.38	4.33	3.44	2.50	3.60
V078	S22	19	E	1	16	17^	3.13	3.89	3.33	2.13	4.20
V079	S23	19	E	4	16	16^	3.63	3.56	3.78	2.88	3.90
V091	S24	21	E	3	15	4	3.13	3.22	3.67	1.75	3.40
V087	S25	18	E	4	17	17^	3.63	4.11	2.56	2.88	3.90
V001	S2	19	I	4	9	12	2.50	3.89	2.33	2.13	3.50
V004	S3	20	I	3	6	18^	2.13	3.22	3.89	3.38^	3.90
V008	S5	19	I	0	7	20^	2.50	3.78	2.75	4.38^	4.10
V013	S6	19	I	2	6	13^	2.00	3.44	3.00	2.63	4.10
V053	S13	19	I	4	9	17^	2.88	3.11	3.00	3.63^	3.30
V065	S15	19	I	5*	6	11	1.25	2.56	3.89	2.88	2.60
V066	S16	19	I	4	6	14^	2.88	3.67	2.89	2.50	3.50
V072	S17	19	I	3	5	19^	2.25	4.11	3.00	2.88	3.30
V071	S18	19	I	5*	10	18^	2.75	2.38	2.22	3.86^	2.78
V074	S19	19	I	1	10	18^	2.88	3.78	2.78	2.88	3.10
V077	S20	19	I	2	3	15^	1.50	3.22	4.00	2.63	3.30
V085	S26	18	I	5*	6	11	2.25	4.00	2.56	2.38	2.80
V090	S27	18	I	2	9	13^	2.50	3.78	3.11	3.25^	3.00
V086	S28	18	I	5*	7	9	2.88	4.11	3.67	2.75	3.40

Notes:

1. Categorial extraversion traits (E – extrovert, I – introvert)
2. EPI scores (L – lie, E – extraversion trait, N – neuroticism trait)
3. BFI scores (E – extraversion trait, A – agreeableness trait, C – conscientiousness trait, N – neuroticism trait, O – openness trait)
4. Caret (^): Neurotic score > 12 in EPI, or ≥ 3 in BFI
5. Asterisk (*): EPI's lie score ≥ 5
